# Oak displays common local but specific distant gene regulation responses to different mycorrhizal fungi

**DOI:** 10.1186/s12864-020-06806-5

**Published:** 2020-06-12

**Authors:** Marie-Lara Bouffaud, Sylvie Herrmann, Mika T. Tarkka, Markus Bönn, Lasse Feldhahn, François Buscot

**Affiliations:** 1 German Centre for Integrative Biodiversity Research (iDiv) Halle-Jena-Leipzig, Deutscher Platz 5e, D-04103 Leipzig, Germany; 2 Department of Soil Ecology, UFZ–Helmholtz Centre for Environmental Research, Theodor-Lieser-Str. 4, D-06120 Halle/Saale, Germany

**Keywords:** *Quercus robur*, Local and distant effects, EMF interaction, OMF interaction, RNA-seq

## Abstract

**Background:**

Associations of tree roots with diverse symbiotic mycorrhizal fungi have distinct effects on whole plant functioning. An untested explanation might be that such effect variability is associated with distinct impacts of different fungi on gene expression in local and distant plant organs. Using a large scale transcriptome sequencing approach, we compared the impact of three ectomycorrhizal (EMF) and one orchid mycorrhizal fungi (OMF) on gene regulation in colonized roots (local), non-colonized roots (short distance) and leaves (long distance) of the *Quercus robur* clone DF159 with reference to the recently published oak genome. Since different mycorrhizal fungi form symbiosis in a different time span and variable extents of apposition structure development, we sampled inoculated but non-mycorrhizal plants, for which however markedly symbiotic effects have been reported. Local root colonization by the fungi was assessed by fungal transcript analysis.

**Results:**

The EMF induced marked and species specific effects on plant development in the analysed association stage, but the OMF did not. At local level, a common set of plant differentially expressed genes (DEG) was identified with similar patterns of responses to the three EMF, but not to the OMF. Most of these core DEG were down-regulated and correspond to already described but also new functions related to establishment of EMF symbiosis. Analysis of the fungal transcripts of two EMF in highly colonized roots also revealed onset of a symbiosis establishment. In contrast, in the OMF, the DEG were mainly related to plant defence. Already at short distances, high specificities in transcriptomic responses to the four fungi were detected, which were further enhanced at long distance in leaves, where almost no common DEG were found between the treatments*.* Notably, no correlation between phylogeny of the EMF and gene expression patterns was observed.

**Conclusions:**

Use of clonal oaks allowed us to identify a core transcriptional program in roots colonized by three different EMF, supporting the existence of a common EMF symbiotic pathway. Conversely, the specific responses in non-colonized organs were more closely related to the specific impacts of the different of EMF on plant performance.

## Background

The roots of numerous ecologically and economically important forest trees of the *Pinaceae*, *Fagaceae*, *Betulaceae*, *Dipterocarpaceae*, *Myrtaceae* and *Salicaceae* live in symbiosis with highly diverse ectomycorrhizal fungi (EMF) of the Basidiomycota and Ascomycota. In these mutualistic associations, the EMF transfer nutrients to the plant and receive photosynthetically derived sugar [1]. It is thought that EMF evolved repeatedly from saprophytic fungi [2] and that this evolutionary pattern is reflected by important ecological and genetic diversity among the EMF [3]. In the last decades, EMF were classified into morphotypes, some traits of which have been used to predict their ecological role on tree performance [4]. However, many EMF cannot be identified at the species level by anatomical description, and their effects on tree growth are sometimes even strain specific [5]. More importantly, root colonization levels and extent of symbiotic apposition structures (i.e. hyphal mantle and Hartig net) are not always correlated with effects on plant growth [6]. Consequently the anatomy of mycorrhiza or the fungal colonization pattern can be considered as a poor predictor of the functional effect of EMF on plants, and comparative studies should rather focus on the functional significance of the mycorrhizal fungal diversity (e.g. [7]).

Most previous transcriptomic analyses of EM symbioses have focused on genome-sequenced plant and fungal species, e.g. *Populus trichocarpa* interacting with *Laccaria bicolor* or *Tuber melanosporum* [8, 9]. Rapid advances in genomics have prompted new projects to sequence genomes of additional mycorrhizal fungi [3] and host trees, e.g., eucalyptus, spruce, oak, chestnut and pine [10–14]. These efforts are generating important resources for comparing the genomic regulation of plants interacting with different EMF partners, and discovering genes of ecological interest.

Establishment of EM symbiosis involves modifications in the development of both partners, including stimulation of roots formation and growth of fungal mycelium [1, 15, 16]. Some impacts such as those on root formation are induced prior to EM formation (early mycorrhizal stage) [17]. Even at these early stages of interaction, different mycobionts can have highly contrasting effects on growth of a same host plant species [18, 19].

Ectomycorrhizal symbiosis leads to huge modifications of plant and fungal gene expression, as shown by transcriptomic analyses of the partners in various plant/fungus associations [20–23]. Functional annotation of differentially expressed genes (DEG) has revealed similar plant gene expression patterns in roots colonized by different EMF, particularly for genes involved in plant cell wall modifications and nutrient transport. This suggests that similar plant metabolic pathways were activated during the parallel evolution of EM symbioses involving different fungal clades, although there is no evidence of a common symbiotic signalling pathway in EM associations [24]. However, most of such gene expression studies have focused on local effects within the mycorrhizal roots and few have considered short distance effects (on gene regulation in non-colonized roots) or long distance effects (in leaves) [25–28], although mycorrhizas influence the physiology of whole plants [29, 30].

The aim of this study was to elucidate how EMF with different evolutionary histories influence gene regulation in *Quercus robur* L. locally and in plant organs at both short and long distances to the colonized roots. We formulated three hypotheses. First, a core set of plant genes is locally regulated by all inoculated fungi, but a larger set of common core genes directly involved in the mycorrhizal symbiosis is locally regulated in a similar manner in associations with the different EMF. Second, the short and long distance plant responses is more specific to each inoculated EMF species, reflecting the variations in their specific effects on plant growth. Third, the phylogeny of the fungi is, at least locally correlated with changes in gene expression patterns induced in the host plant, i.e. phylogenetically related fungal taxa may induce a more similar plant regulation pattern than more distantly related EMF. To test these hypotheses, we inoculated genetically identical saplings of *Q. robur* clone DF159 (the TrophinOak platform: www.trophinoak.de) separately with four basidiomycetes: three EMF and one orchid mycorrhizal fungus (OMF). Two of the EMF, *Paxillus involutus* ATCC200175 and *Pisolithus microcarpus* 441, are members of the *Boletales* formally used for molecular studies on mycorrhiza formation with *Betula* and *Eucalyptus*, respectively [21, 31]. The other EMF, *Laccaria bicolor* S238N, a member of the *Agaricales*, has been widely investigated in EM associations with the model tree species *Populus trichocarpa* [32]. The three EMF belong to taxa shown to form fully developed mycorrhiza on DF159 [18]. The OMF, *Serendipita vermifera* MAFF 305830, belongs to the *Sebacinales* clade B, whose members were originally described as orchid mycorrhizae, but recent DNA studies have shown that they are able to form a broader spectrum of mycorrhiza [33–35]. We have found no reports of this OMF forming ectomycorrhizas with oaks [36], but it stimulates growth of several plant species, such as *Arabidopsis thaliana*, *Panicum virgatum* and *Nicotiana attenuata* without forming typical mycorrhizal structures [37–39]. Herrmann and Buscot [40] and Frettinger et al. [41] have shown that gene regulation patterns in the oak clone DF159 are largely similar in pre-mycorrhizal roots and fully developed EM. Hence, we compared responses of DF159 oak saplings to inoculation with the four fungi under high humidity conditions to avoid the formation of full mycorrhizas as described by Herrmann et al. [42]. This experimental strategyavoided biases due to comparing mycorrhizas at different stages of differentiation with a non-mycorrhiza forming OMF.

Transcriptomes of the saplings were analysed by Illumina sequencing, reads were aligned against the recently sequenced oak genome [12], and 12 of the differentially expressed transcripts were validated by qRT- PCR. Local, short distance and long distance responses to the fungi were then compared by analysing transcriptomic changes in colonized roots, non-colonized roots and leaves, respectively.

## Results

### Effects of fungal inoculation on oak growth

After 13 weeks of co-culture, total plant fresh weight and total root length were significantly enhanced by the three EMF *P. microcarpus, P. involutus* and *L. bicolor* (Additional file 1: Fig. S1a,c), but not affected by the OMF *S. vermifera*. The root/shoot ratio (R/S) was increased under the *P. microcarpus* and *P. involutus* treatments but not affected by *L. bicolor* and *S. vermifera* inoculations (Additional file 1: Fig. S1b). Total leaf area was significantly increased by treatments with *P. microcarpus* and *L. bicolor*, but not by *P. involutus* or *S. vermifera* treatments (Additional file 1: Fig. S1d).

### Oak and fungal read alignments

Illumina RNAseq was performed on colonized roots, non-colonized roots and leaves of control plants (not inoculated) and plants inoculated with the four fungi. Between 26 and 32 million reads were successfully aligned on the oak genome and assigned to genes for colonized root, non-colonized root and leaf samples (Additional file 2: Figure S2).

To assess the degree of fungal mycelium association in colonized (local) and optically non-colonized (short distance) roots of oak, the RNA-seq reads from the two root types were aligned on the genomes of the interacting fungi. In colonized roots, 11% of reads aligned to *L. bicolor* and 13.5% to *P. involutus genome*, but only 3.8% to *P. microcarpus* and 1.2% to *S. vermifera*. As expected, the alignment rates were markedly lower in non-colonized roots, with 2.9, 1.2, 0.6 and 0.9% of reads for *L. bicolor*, *P. involutus*, *P. microcarpus*, and *S. vermifera*, respectively.

### Fungal symbiosis-related genes are expressed in colonized roots

Ectomycorrhizas were not detected in the densely colonized roots, but the levels of read alignment on *L. bicolor* and *P. involutus* genomes represented 11 and 13%, of total reads, respectively. Since this corresponds to more than four million reads per sample, fungal gene expression between colonized roots and free living mycelium was compared. In total 962 genes were up- and 1132 down-regulated in *L. bicolor* and 499 genes were up- and 348 down-regulated in *P. involutus* (Additional file 3: Table S1).

Several highly up-regulated fungal genes encoded mycorrhiza-induced small secreted proteins (MiSSPs) that have been implicated in ECM formation (Additional file 3: Table S1 and Additional file 4: Fig. S3). The 40 most up-regulated genes for *L. bicolor* encoded six MiSSPs: LbMiSSP7, LbMiSSP7.61, LbMiSSP8, LbMiSSP11.4 and LbMiSSP17, with up-regulation levels of 80-fold (*LbMiSSP7.61*) to more than 7000-fold (*LbMiSSP8*). Other highly up-regulated genes in *L. bicolor* included *cysteine proteinase inhibitors, ammonium transporters, glutamate dehydrogenase, zinc dependent metalloprotease, major facilitator superfamily transporters* and *carbohydrate-binding protein*. In addition, *L. bicolor* transcripts encoding carbohydrate active enzymes were up-regulated, including endoglucanases from glycoside hydrolase family 5 (Lb319772) and family 12 (Lb385634, Lb320398 and Lb477020), as well as glycoside hydrolase family 28 polygalacturonase (Lb612983). As observed with *L. bicolor*, the most highly root contact-induced genes in *P. involutus* encoded small secreted polypeptides, such as *Pi167671* with 105-fold and *Pi20703* with 46-fold up-regulation. Genes specifically and strongly up-regulated in *P. involutus* encoded *hydrophobin, protein kinase, phosphatidylserine decarboxylase, acetate transporter, glutaredoxin, thioredoxin disulfide reductase and cytochrome P450 monooxygenase*. Carbohydrate active enzyme genes were not up-regulated in *P. involutus.*

### Core DEG in EMF-colonized oak roots

**Fig. 1:**
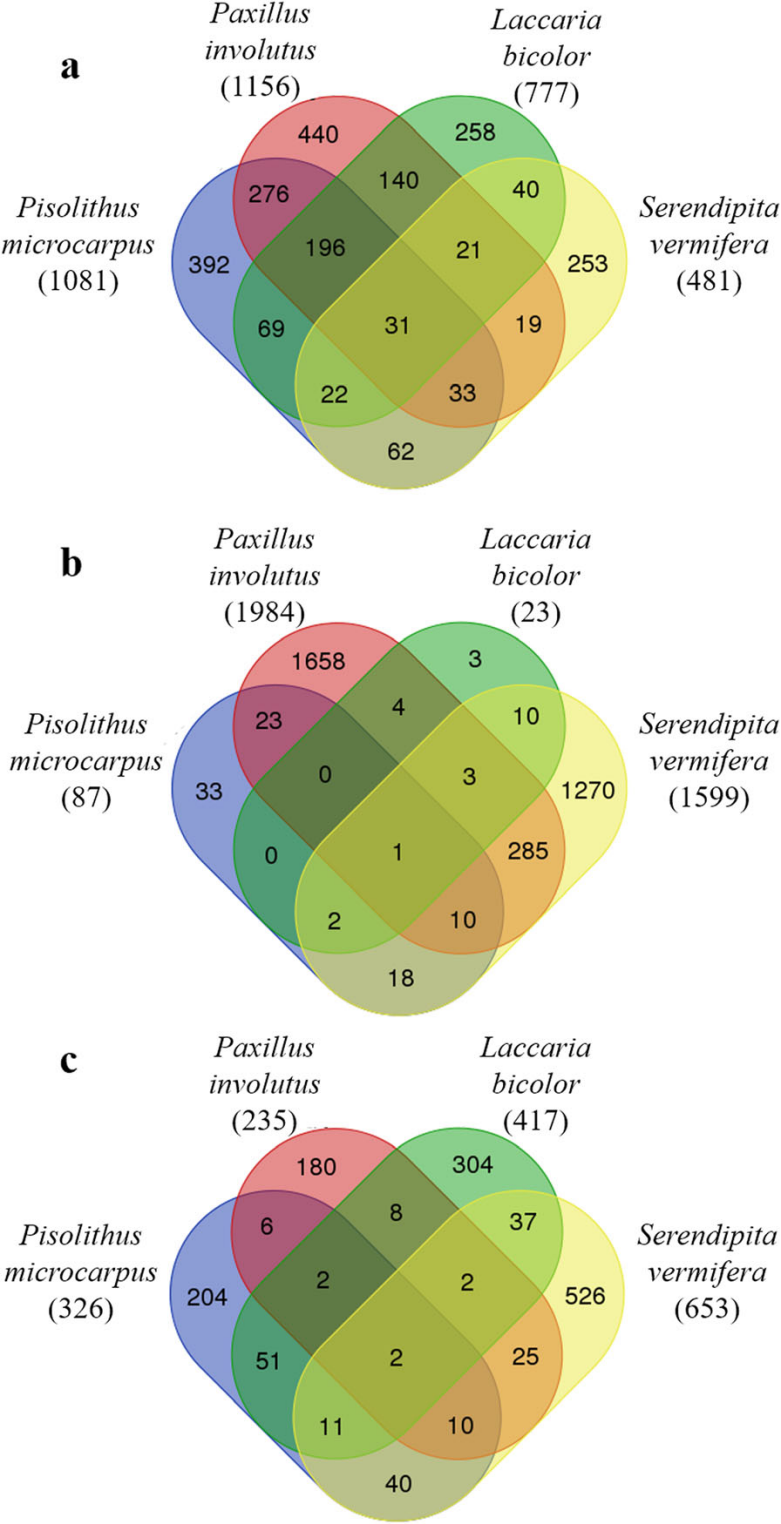
Venn diagrams showing numbers of differentially expressed genes in oak after inoculation with mycorrhizal fungi. Differential expression thresholds of RNA sequencing data were at least 2-fold difference to control (no fungus) and a Benjamini-Hochberg adjusted *P* < 0.01. Numbers in brackets indicate the total number of differentially expressed genes under each treatment. (**a**) colonized oak roots, (**b**) non-colonized oak roots, and (**c**) leaves after inoculation of *P. microcarpus*, *P. involutus*, *L. bicolor* and *S. vermifera*

In total, 2252 genes were differentially expressed, relative to controls, under the four fungal treatments: 1081, 1156, 777 and 481 in roots colonized by *P. microcarpus*, *P. involutus*, *L. bicolor* and *S. vermifera*, respectively (Fig. 1a). A core set of 31 genes was differentially expressed in colonized roots, relative to controls, under all four inoculation treatments (Fig. 1a, Table 1). Most (23) of these core genes were up-regulated, and related to cell-wall organization, including inositol oxygenase and trehalose-phosphate synthase encoding genes. Seven genes of the core set were down-regulated transporter and photosynthesis-related genes. Finally one gene of the core set was a polygalacturonase-encoding gene (*Qrob_P0350390.2*), which was down-regulated by the EMF, but up-regulated by the OMF (Table 1).

**Table 1:**
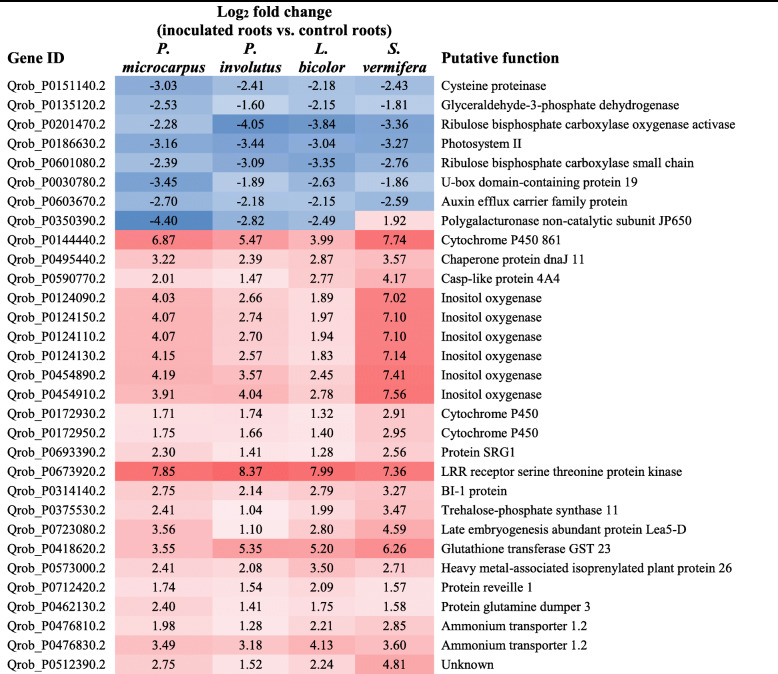
Thirty-one core genes showing differential expression in colonized roots after inoculation by the mycorrhizal fungi *P. microcarpus*, *P. involutus*, *L. bicolor* and *S. vermifera*. Genes with at least 2-fold difference relative to fungus-free controls and a Benjamini-Hochberg adjusted *P*<0.01 were selected

In accordance with our first hypothesis, a stronger common response of 196 core DEG was observed in colonized oak roots when the comparison was restricted to the three EMF treatments (Fig. 1a, Table 2 and Additional file 5: Table S2). Clearly corresponding to a general plant response to EMF, all of these 196 core DEG were regulated in the same direction by the three EMF. Most of them (87%) were down-regulated, including genes encoding proteins involved in carbon metabolism, defense responses, phenolic pathways and transport (Table 2). Due to the well-known impact of biotic interaction on plant hormone equilibrium, phytohormone-related genes were checked for all interactions. Genes involved in auxin biosynthesis, transport and responses were globally affected, relative to controls, by interactions with the three EMF, but not by the OMF *S. vermifera*. In contrast, ethylene- and gibberellic acid-related genes were differentially regulated in both the EMF- and OMF-treated roots (Additional file 6: Table S3).

**Table 2:**
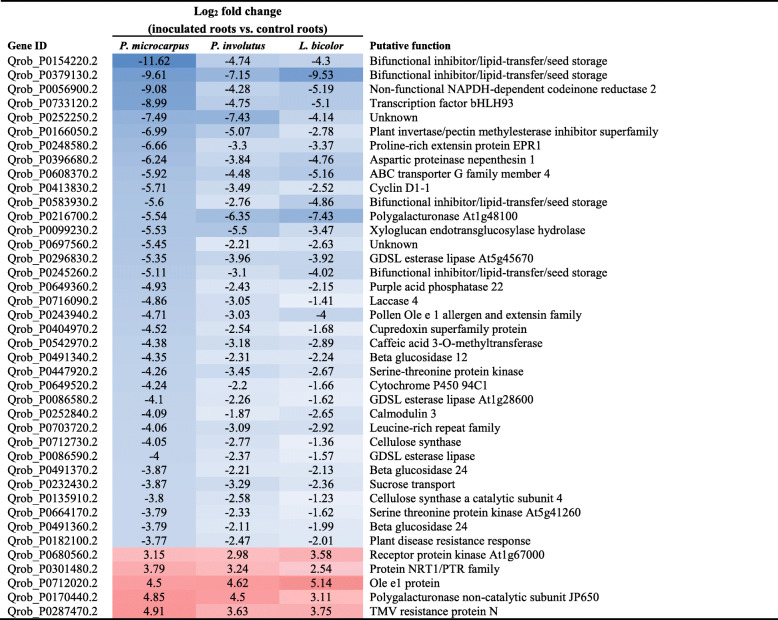
Forty genes showing the largest differential expression in colonized roots after inoculation by the EMF *P. microcarpus*, *P. involutus and L. bicolor*. Genes with at least 2-fold difference relative to fungus-free controls and a Benjamini-Hochberg adjusted *P* < 0.01 were selected

### Fungus-specific gene expression patterns identified in non-colonized oak roots

In non-colonized roots, 3320 genes were identified as differentially expressed, relative to controls, under the four fungal treatments. Notably, the number of DEG strongly varied, depending on the inoculated fungus. 1984 and 1599 genes were differentially expressed in non-colonized roots of plants inoculated with *P. involutus* and *S. vermifera,* respectively, but only 87 and 23 in non-colonized roots inoculated with *P. microcarpus* and *L. bicolor*, respectively (Fig. 1b). Moreover, only one core gene regulated in non-colonized roots under all four treatments was detected. Transcripts of this gene, *Qrob_P0673920.2*, encoding a leucine-rich receptor serine threonine kinase, were among the most strongly enriched (log_2_ fold change between 7.58 and 8.45) in all four interactions (Additional file 7: Table S4). In non-colonized roots, most of the DEG were linked to the interaction with just one fungus, few overlaps between three or even two inoculation treatments were observed. Regarding the EMF, no gene was differentially expressed in non-colonized roots under treatments by all three EMF. In accordance with the second hypothesis, that the fungi would induce species-specific ‘short-distance’ responses in non-colonized roots of host plants, the genes with the highest fold-changes under each of the four treatments had different functions. For example, the treatments with *P. microcarpus* and *P. involutus* induced down-regulation of genes related to phytohormone (cytokinin and ethylene) biosynthesis and reduction of ferric iron*,* respectively (Additional file 7: Table S4). Less specifically, a global response concerning the plant hormone-related genes, particularly genes involved in auxin pathways, was detected in non-colonized roots under both *P. involutus* and *S. vermifera* treatments (Additional file 6: Table S3). However, in accordance with the specificity hypothesized, this auxin response was coupled with modifications in gibberellic acid (Gibberellic acid methyltransferase, Gibberellin 20 oxidase, Gibberellin 2-beta-dioxygenase and Ent-kaurene) and ethylene (1-aminocyclopropane-1-carboxylate oxidases) pathways only in the presence of the OMF *S. vermifera*.

The specificity of responses observed in DEG in non-colonized roots were also reflected in GO-term enrichment patterns (Additional file 8: Table S5), in both the numbers of enriched GO-terms and the directions of regulation (up or down) of genes associated with given GO-terms. No GO-term was enriched among the few DEG associated with the *L. bicolor* treatment, and only two (*photosynthesis, light harvesting* and *galactose metabolic process*) were associated with the *P. microcarpus* treatment. In contrast, 25 GO-terms were enriched in non-colonized roots after *P. involutus* inoculation. These included GO-terms related to the regulation of transcription among the up-regulated genes, and GO-terms related to cell-wall organization and defense responses among the down-regulated genes.

In contrast, the *S. vermifera* treatment led to up-regulation of defense response genes in non-colonized roots, as revealed by enrichment in *defense response* and *response to biotic stimulus* GO-terms. Strong expansion of genes related to biotic interactions has been detected in the oak genome, particularly those encoding nucleotide-binding site leucine-rich repeat (NB–LRR) proteins and receptor-like kinases (RLK), for which 95 and 55% expansion rates have been estimated, respectively [12]. Thus, we focused on these groups of potential defense-related genes. Many of these plant disease resistance genes were differentially expressed in non-colonized roots under the fungal treatments. They included 165 of the 1091 (14.8%) *NB-LRR* and 256 of the 1247 (20.5%) *RLK* genes (Additional file 9: Fig. S4 and Additional file 10: Table S6). Most of these plant genes responded in a fungus-specific manner. Interestingly, even though the proportion of RLK genes was similar with *P. involutus* and *S. vermifera* (5.5 and 5.6%, respectively), under the *S. vermifera* treatment, genes of *LRR-RLK* subgroup XIIa, which include receptors known to participate in responses to microbial infection, as well as lectin receptor kinases known for their role in plant defense and immunity, were specifically differentially expressed in non-colonized roots (Additional file 10: Table S6).

### Confirmation of fungus-specific long distance responses in leaves

In leaves, 1408 DEG were identified, relative to controls, under the four fungal treatments (Additional file 11: Table S7). For 12 leaf DEG in plants inoculated with *L. bicolor,* we found a good agreement between estimates of the differential expression levels obtained by qRT-PCR and transcriptome analysis, confirming the overall value of the RNA-seq data (Additional file 12: Fig. S5). Numbers of DEG of the same order of magnitude (235 to 417) were obtained after inoculation with all three EMFs, while the OMF (*S. vermifera*) treatment resulted in the highest number (653). Only two genes were differentially expressed in leaves under all four fungal treatments (Fig. 1c), one encoding a metacaspase 1 (Qrob_P0407070.2) and the other a superoxide dismutase copper chaperone (Qrob_P0502610.2). Even under the three EMF treatments, only two more common DEG were identified in leaves, one encoding a sucrose synthase (Qrob_P0403240.2) and the other an F-box kelch-repeat protein (Qrob_P0245400.2). Similarly, the only consistent pattern of changes in expression of phytohormone-related genes in leaves under all three EMF treatments was the down-regulation of genes involved in abscisic acid degradation. However higher specificity in responses to the four fungi was observed in expression patterns of the other hormone-related genes (Additional file 6: Table S3). In leaves, the high specificity of changes induced by the four fungi was confirmed by GO-term enrichment analyses, which revealed specific modifications to plant functions induced by each fungus at long distance (Table 3). *Laccaria bicolor* inoculation induced enrichment of GO-terms associated with plant cell wall organization including *lignin catabolic process*, *microtubule-based process* and *plant-type cell wall organization*. In contrast, the two other EMF inoculations led to enrichment of only one GO-term, *photosynthesis and light harvesting* with *P. involutus* and *oxidation-reduction process* with *P. microcarpus*. Finally, *S. vermifera* inoculation induced enrichment of GO-terms related to cell wall biogenesis. In conclusion, the oak displayed highly fungus-specific responses at long distance in the leaves, demonstrating the specificity of the distant impacts of the three EMF on the host tree.

**Table 3:**
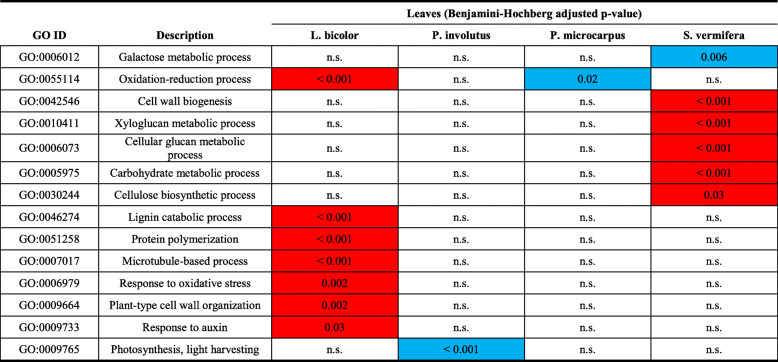
GO-term enrichment analysis (Biological processes) performed on leaves for the different treatments. Values correspond to Benjamini-Hochberg adjusted *p*-values obtained using GOseq [43], in red for up-regulated GO-terms and in blue for down-regulated GO-terms. n.s.: not significant

As characteristic distant plant responses were detected for each fungus, we compared the DEG to mobile mRNAs in *A. thaliana* listed by [44–47] to identify whether the long distance effects were correlated with potential mobile mRNAs. Closest homologs in *A. thaliana* of about 14–23% of the genes differentially expressed in both roots and leaves under each inoculation treatment correspond to putative mobile mRNAs (Additional file 13: Table S8). Up- or down-regulation of most of these putative mobile mRNAs was associated with a specific mycorrhizal fungus, and contributed to the previously described specificity of distant plant responses. Under *P. microcarpus* and *L. bicolor* treatments, the DEG among the putative mobile mRNAs were related to cell wall processes, whereas under *P. involutus* and *S. vermifera* treatments they related to photosynthesis and defense responses, respectively.

### Is fungal phylogeny a good predictor of induced plant regulation patterns?

**Fig. 2:**
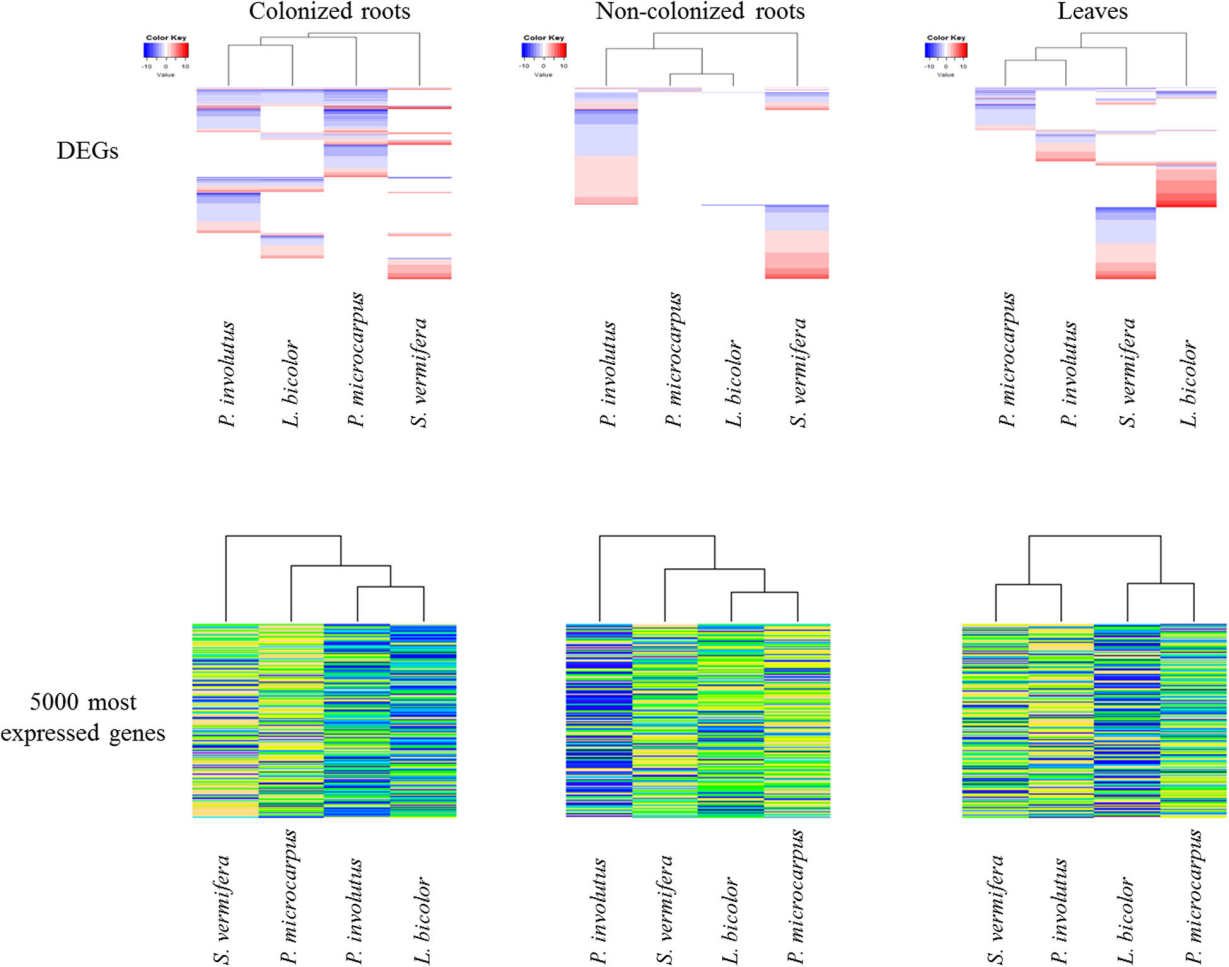
Heatmaps of DEG and expression levels of the 5000 most highly expressed genes. Genes are shown in colonized roots, non-colonized roots and leaves

The phylogeny of the four studied fungi was compared to the changes they induced in the expression patterns. The analysis was based on the DEG data and expression levels of the 5000 most highly expressed genes in each of the three considered plant parts. In colonized roots, the changes induced by the three EMF were more similar to each other than those induced by the OMF *S. vermifera*, according to heatmap-based clustering of data for both the DEG and 5000 most highly expressed genes (Fig. 2). However, the EMF phylogeny was not congruent with the observed clustering, as heatmaps of changes induced by *L. bicolor* (Agaricales) and *P. involutus* (Boletales) clustered together, while the heatmap of changes induced by the other EMF member of the Boletales, *P. microcarpus*, was more distant. In non-colonized roots, the three EMF induced closer DEG profiles than the OMF, but again the similarity of changes induced by the EMF did not correlate with their phylogenetic closeness. Clustering based on the 5000 most highly expressed genes did not confirm this pattern of responses in non-colonized roots, as the changes in gene expression induced by *P. involutus* differed most strongly from those induced by the other fungi (two other EMF and an OMF). In leaves, the clustering based on DEG and the 5000 most highly expressed genes showed that the three EMF did not induce more similar changes than the OMF (Fig. 2), confirming thereby the specificity of the distant plant responses to the four tested species of mycorrhizal fungi.

## Discussion

Formation of ectomycorrhizas on roots by different EMF requires variable timespans after inoculation, and extent of apposition structures is heterogeneous and their development is not synchronous even on root systems developed in microcosms [18]. Consequently, it cannot be expected that different fungi inoculated on a same plant species will reach similar colonization intensities and rates at a given sampling time, even in a standardized culture system as the one with clonal oak cuttings used in this work [17]. In addition the presented experiment included a *Serendipita* non able to differentiate EM. For these reasons our experiment deliberately analyzed plants in interactions without EM differentiation, knowing that in the used oak system, inoculation alone exerts typical symbiotic effects on development and physiology [42] as well as on gene regulation [41] of plants, which are similar to the one observed after complete formation of ectomycorrhizas. This functional impact on the plant in absence of symbiotic structures was confirmed for the EMF tested here by their influence on plant growth and fresh weight. Additionally, in this experiment, the different proportions of fungal and plant transcripts found in colonized vs. non-colonized roots allowed us to confirm that at least for two out of the three EMF (*P. involutus* and *L. bicolor*), the fungi markedly colonized host roots locally, close to the inoculation points in the microcosms. Despite a weaker colonization revealed by its transcript analysis, the third EMF *P. microcarpus* had significant effects on plant growth, which have already been described for other fungi [1, 48].

### Fungal response to oak roots includes *MiSSP* and *plant cell wall degrading enzyme* gene expression

Although sampling of the plant material was performed before mycorrhiza differentiation, *L. bicolor* genes related to the full establishment of the symbiosis were up-regulated in the colonized roots. Of the mycorrhiza induced small secreted polypeptide (MiSSP) gene products of *L. bicolor*, *MiSSP7* induction leads to alterations of the plant transcriptomic profile and modification of plant immunity during colonization [32], LbMiSSP8 is involved at early stages of ectomycorrhiza formation [49], and MiSSP7.6 has been implicated in the modulation of plant gene expression during root colonization [50]. Interestingly, *MiSSP7* is induced by poplar root exudates prior to root colonization process, and the abundance of MiSSP7.6 transcripts increases similarly in ectomycorrhizas and in the extramatricial mycelium close to the roots, indicating that direct physical contact with roots is not required to trigger *MiSSP7* or *MiSSP7.6* induction [50, 51]. These observations are in line with ours on *MiSSP* gene induction prior to ectomycorrhiza formation. The abundance of plant cell wall degrading enzyme transcripts of *L. bicolor* also increased on the colonized roots. The survey of ectomycorrhizal fungal genomes by Kohler et al. [3] demonstrated substantial losses in such genes as compared to saprophytic fungi, but the cell-wall-degrading enzyme genes are expressed in a limited manner during ECM establishment. Zhang et al. [52] showed that Lb319772 endoglucanase, one of the induced genes on colonized oak roots, is involved in plant cell wall modifications during symbiosis formation in poplar.

### Different EMF induced a common local core transcriptional program in colonized roots

In accordance with our first hypothesis that a common set of genes related to symbiosis are regulated similarly by different EMF, a much broader range of plant core genes were found to be induced by all three EMF species within colonized roots compared to the number of genes common to the four mycorrhizal treatments. The proportion of DEG that accounted for these ‘core genes’ is similar to proportions observed in chickpea colonized by rhizobia, pathogenic oomycetes or arbuscular mycorrhizal fungi [53]. However, our study revealed several core genes that were not previously identified in other plant-mycorrhizal fungus interaction studies. These include genes encoding inositol oxygenase, an enzyme involved in cell wall biosynthesis [54], which is consistent with its likely participation in establishment of the apposition structure between plant and fungal walls in the Hartig net of mycorrhizae. Most of the core genes that were differentially expressed under all three EMF treatments (including genes encoding components of defense response and phenolic pathways) were down-regulated. This is consistent with the attenuation of cortical host cells’ defenses in roots that promotes fungal penetration [22, 23, 55]. At this local site of interaction, a modification of the auxin pathways was observed in the host plant during interaction with the three EMF but not with the OMF *S. vermifera*. This is congruent with auxin’s major role in EMF-induced root differentiation [56–58]. It is also consistent with increases in root/shoot ratios previously recorded following inoculation of the same oak clone (DF159) with the EMF *Piloderma croceum* [42], and may therefore be responsible for the increases we observed in root system lengths of plants inoculated with each of the three EMF. In addition, comparison of changes induced by the different EMF partners during interaction with genetically identical oak saplings enabled identification of further locally regulated core genes. These include genes previously reported to be differentially expressed in oak mycorrhizal roots [23] such as genes encoding for *laccase, cellulose synthase, polygalacturonase* and *xyloglucan endotransglucosylase hydrolase,* but also other genes with potential role in EM establishment such as a *plant disease resistance response protein* and a *receptor protein kinase* encoding gene.

The detected plant core response can reflect a common symbiotic pathway [59] activated by different EMF. This is supported by our finding of fungal symbiosis-related genes activated in *L. bicolor* and *P. involutus* hyphae during root colonization. Formation of ECM symbiosis requires lineage-specific genes encoding small secreted proteins that were evidenced in the fungal transcripts of both *L. bicolor* and *P. involutus*. Further found genes like *hydrophobin, phosphatidylserine decarboxylases, glutaredoxin,* and *cytochrome P450 monooxygenase* have also previously been reported to be up-regulated in ECM tissues [60, 61], confirming that even without differentiation of mycorrhiza a symbiotic plant-fungus dialog was already set up in the colonized roots.

### Distant plant responses are highly fungus-specific

The second hypothesis, that short and long distance responses of plants to specific EMF may differ was validated in terms of plant growth responses, numbers of DEG and the metabolic pathways affected. Notably, we observed two patterns of short-distance responses, as few DEG were associated with the *P. microcarpus* and *L. bicolor* treatments, but high numbers were associated with the *P. involutus* and *S. vermifera* treatments. The smaller short-distance effects of *P. microcarpus* and *L. bicolor* on gene expression was not due to a general lack of host plant responses, as these interactors strongly affected gene expression in leaves. Moreover, despite only changing the expression of a few plant genes in non-colonized roots, *P. microcarpus* and *L. bicolor* had strong morphological effects, including more than 400 and 250% increases in root length and total plant fresh weight, respectively. Inoculation with *P. involutus* had strong effects on both host plant growth and gene expression in non-colonized roots. The DEG included genes involved in auxin and jasmonic acid pathways, which are known to play roles in both root growth [62] and formation of ectomycorrhiza and arbuscular mycorrhiza [16, 32, 58, 63]. In contrast, the short-distance responses induced by the non-EMF *S. vermifera* concern the regulation of numerous genes, particularly up-regulation of genes involved in defense responses, which is consistent with the probably non-symbiotic relationship between this OMF and the oak. As the high humidity conditions of our experiment hamper formation of ectomycorrhiza [42], it was not suitable to explore the potential of the OMF to form EMF. However, contrarily to the three tested EMF species, the OMF had no impact on the plant growth.

Large-scale comparative molecular studies on long-distance plant responses to EMF are scarce. A study focusing on defense-related genes in shoots of *Medicago truncatula* in presence of three arbuscular mycorrhizal species has been reported [28]. Here we show that more general long-distance plant responses, in leaves, are clearly induced by inoculation with EMF and even with an orchid mycobiont, and that the responses are strongly dependent on the fungal partner. The specific changes observed in expression patterns in leaves included up-regulation of genes related to cell wall modification-related genes following inoculation with *L. bicolor*, and the identification of putative mobile mRNAs related to cell wall organization among products of DEG detected under treatment with this fungus is consistent with the enhancement of aboveground plant growth. In contrast, no significant increase in leaf area occurred after *P. involutus* inoculation, but here modification in the regulation concerned genes involved in carbohydrate allocation to roots. Thus, *P. involutus* seems to influence energy storage, putatively changing the balance between soluble sugar and starch. Induction of an increase in sucrose contents of *Populus euphratica* leaves by *P. involutus* has been previously described, even in the apparent absence of mycorrhiza or changes in leaf growth [64]. This suggests that *P. involutus* has evolved a more specific strategy to benefit from plant carbohydrates than the two other studied EMF.

### Particularity of the non-host interaction with *S. vermifera*

In contrast to the EMF, *S. vermifera* induced up-regulation of genes encoding chitinases, and the induction of several genes encoding beta-1.3-glucanases, ethylene responsive transcription factors, caffeic acid methyltransferases and flavonol synthases, some of which were also identified as putative mobile mRNAs. The defense responses were detected both at short distance in non-colonized roots and long distance in leaves, and these observations suggest that the OMF induces general defense responses and secondary metabolism pathways, perhaps in a similar manner to the reported induction of oak defenses against the biotrophic pathogen *Microsphaera alphitoides* induced by a mycorrhiza helper bacterium [65]. The OMF generally form friendly associations with their hosts without eliciting defense responses [66]. However, an interesting outcome of our comparison is that *S. vermifera* seems to elicit responses in the oak saplings, which are different from their responses to true EMF. The up-regulation of defense-related genes suggests that *S. vermifera* may induce a non-host response in oak, including up-regulation of large proportions of the NB-LRR and RLKs genes identified in Plomion et al. [12] in non-colonized roots, particularly lectin receptor kinases and LRR-RLKs known to induce defense responses and mediate plant immunity [67, 68]. Such defense response might be related to oak being a non-host of *S. vermifera*. Although other *Sebacina* species are ectomycorrhizal in oaks [36], *S. vermifera* does not colonize oaks in nature, and thus the *S. vermifera* isolate used in the present study might show an incompatibility reaction to the oak clone.

### Fungal phylogeny is not correlated to induced plant responses

Contrary to the third hypothesis, fungal phylogeny was not a good predictor of oak gene expression patterns. As expected, the transcriptomic changes induced by the OMF in the host plants’ colonized roots differed from those induced by the three EMF. However, the differences did not agree with the phylogenic distances between the three EMF, as the patterns of responses to *P. involutus* (Boletales) and *L. bicolor* (Agaricales) were more similar to each other than to those induced by the other member of the Boletales, *P. microcarpus*. Interestingly, we found that the phylogenic distances between the tested fungal taxa were less strongly connected to transcriptomic changes in the distant plant parts than in the infected roots. In this respect, there was a clear distinction between the three EMF and the OMF in infected roots, but not in non-colonized roots and leaves. These results are consistent with the common traits of EMF apposition tissues (i.e. hyphal mantle and Hartig net within EM roots), but sometimes markedly different effects on the growth of the whole host plant, due to specific patterns of distant gene activation. EMF have evolved independently from multiple fungal phyla, and it has been estimated that more than 80 lineages exist [69]. Phylogenetic and genomic analyses suggest that they convergently evolved from white- and brown-rot fungi and from soil saprotrophs [2, 70], and even within the *Boletales*, the ectomycorrhiza formation capacity of *Paxillus* and *Pisolithus* spp. may have emerged independently [3]. If so, clear associations between evolutionary relationships of EMF and the changes they induce in plant expression patterns would only exist among taxa of the same ectomycorrhizal lineage or which are derived from the same ancestor [70].

## Conclusion

Our study allowed the first molecular-level comparison of local and distant effects of three EMF and one OMF on different parts of a genetic identical host tree (*Q. robur* clone DF159), with reference to the recently released oak genome. Through comparative transcriptome analysis, we identified a core set of EMF-induced genes in the oak at the local site of interaction. This supports the existence of common EMF symbiotic pathways [59], despite dissimilar “symbiotic toolkits” of the EMF previously reported [3]. Conversely, in non-colonized roots and leaves, effects were strongly fungus-specific and did not correlate with their phylogenetic relationships. Mycorrhizal inoculation may trigger diverse distant mechanisms like signaling or transfer of metabolites to target organs, which is also supported by the identification of affiliated putative mobile mRNAs known to migrate through the plants. These distant mechanisms vary according to the EMF partner, leading to high heterogeneity of the distant responses, in accordance with evidence that the ectomycorrhizal functional trait evolved independently in distinct fungal lineages [2], resulting in different EMF taxa having different regulatory effects on the same host tree.

## Methods

### Plant and fungal growth conditions

The pedunculate oak clone DF159 (*Quercus robur* L.) was micro-propagated and rooted as described by Herrmann et al. [17]. The four used mycorrhizal fungal strains included the ectomycorrhiza forming fungi *Pisolithus microcarpus* 441, *Paxillus involutus* ATCC200175 and *Laccaria bicolor* S238N, as well as the orchid mycorrhiza forming fungus *Serendipita vermifera* MAFF305830. They were pre-cultivated in Petri dishes on Melin Norkrans Modified agar medium (MMN agar medium [71];) supplemented with 0.1% (w/v) casein hydrolysate (MMNC [17];) in darkness at 20 °C. Fungal inoculum was produced by inoculating a mixture of 750 ml of perlite with 100 ml of MMN liquid medium supplemented with 0.5% (w/v) glucose and 100 ml of a 2–3 weeks old liquid fungal culture produced in MMNC medium, in darkness at 20 °C with shaking at 100 rpm. Rooted micro-cuttings were transferred into closed glass vessels containing 150 ml of the two-week-old inoculum, 150 ml of sterilized perlite and 30 ml of MMN liquid medium with no carbohydrate and tenth**-**strength P and N (MMN1/10 [17];). Non-inoculated control plants were grown in similar glass vessels with 300 ml of sterilized perlite and 50 ml of MMN1/10 liquid medium. Plants were grown at 23 °C with 16 h photoperiod, with a photosynthetic photon flux density of 180 μmol m^− 2^ s^− 1^ during light phases, for 13 weeks.

### Morphological analyses of the oak saplings

At harvest, the oaks at bud rest stage A of the endogenous rhythmic growth [25] were gently lifted from the vessels, perlite was carefully removed with forceps and placed on filter paper for photography. Plants measurements were performed on these photos using WinRhizo and WinFolia pro V2005b (Regent Instruments). Plant growth parameters (fresh weight) were evaluated on the complete plant using a minimum of eight replicates (excepted for saplings inoculated with *L. bicolor*, 6 replicates) and compared using the Kruskal-Wallis test implemented in R software [72]. Between-treatment differences in these parameters were considered significant if *P* < 0.05.

### RNA extractions and Illumina RNA-Seq

For RNA extractions, three types of plant material were harvested: colonized roots represented by short roots with visible, dense (*L. bicolor, P. involutus, P. microcarpus*) or loose (*S. vermifera*) fungal mycelium covering their surfaces; non-colonized roots characterized by short roots without any visible fungal mycelium covering their surfaces, and fully developed source leaves belonging to the last developed shoot flush. An absence of mycorrhizas was controlled by *de visu* observation with dissection microscope on a small set of lateral roots. At harvest, tissues were immediately submerged in liquid nitrogen and the material was stored at − 80 °C. Total RNA was extracted from 50 mg portions of leaf material and 100 mg portions of colonized and non-colonized root material using a MasterPure Plant RNA Purification Kit (Epicentre, Hessisch Oldendorf, Germany). Three biological replicates from single plants were used for non-colonized roots and leaves. For the colonized roots, two (*L.bicolor*, *P. microcarpus* and *S. vermifera*) to three (*P. involutus*) biological replicates from four to six plants were considered. The extracted RNA was treated with DNase I (Fermentas, St Leon-Rot, Germany), and quantified using a NanoDrop spectrophotometer (Thermo Scientific, Passau, Germany) and a Quant-iT RiboGreen RNA Assay Kit (Invitrogen, Darmstadt, Germany). Its quality was checked using a Nano Chip and Bioanalyzer 2100 (Agilent, Böblingen, Germany). cDNA libraries were prepared from the total RNA using Illumina TruSeq RNA Sample Preparation kit v2 and sequenced using a 2 × 100 bp Illumina HiSeq 2000 platform at the Beijing Genomics Institute, Hong Kong, China. Raw sequence data are available in the Sequence Read Archive at the National Center for Biotechnology Information (BioProject accession PRJNA516042).

### Read processing and statistical analysis

Low complexity and low-quality reads were removed with SeqClean http://sourceforge.net/projects/seqclean/files/). Nucleotides with quality score < 20 were removed from the ends of the reads using a custom Java script. Sequences < 50 bp were discarded, as well as sequences lacking paired-end information. Illumina reads were aligned against the haploid oak genome [12] by TopHat2 v.2.1.0 [73] and gene expression levels quantified by featureCounts in the subread package v.1.4.6 [74]. The significance of differences in gene expression was assessed using edgeR v.3.10.2 [75] implemented in the Bioconductor package. Genes were considered differentially expressed when the Benjamini-Hochberg adjusted *P*-value was less than 1% and the log2-fold change of gene expression was greater than 2. Blast2GO [76] was used to classify the genes in Gene Ontology (GO) terms [77]), and GO enrichment analysis was performed with the Bioconductor package GOseq v.1.20.0 [43], and GO-terms were considered enriched when the Benjamini-Hochberg adjusted *P*-value was less than 5%. Differentially expressed genes (DEG) were subjected to blastx search against the NCBI nr and *Arabidopsis thaliana* TAIR databases. Only hits with an E-value of at least <1e-5 were taken into account. Heatmapping was performed using the DEG data and the number of reads of the 5000 most strongly expressed genes using the *heatmap* function in the R software. The list of the nucleotide-binding site leucine-rich repeat (NB–LRR)-related protein genes and receptor-like kinase (RLK)-encoding genes were extracted from the data published by Plomion et al. [12] and compared to the DEGs identified. For analysis of mobile mRNAs, DEG detected in both roots (colonized and non-colonized) and leaves were compared to the list of 2249 known mobile mRNAs of *A. thaliana* identified by [44–47].

To assess how the fungal mycelium contributes to gene expression in colonized and non-colonized roots, reads from the colonized and non-colonized roots samples were aligned using TopHat2 v.2.1.0 [73] on the genomes of the interacting fungi: *L. bicolor, P. involutus, P. microcarpus* and *S. vermifera*. Fungal genomes information was retrieved from Joint Genome Institute’s Mycocosm database [78]. Alignment results of colonized roots showed that from the 40 million reads, the highest mapping rates were for *L. bicolor* (average 4,440,291 reads comprising 11% of input), followed by *P. involutus* (3,717,004, 9.3%), *P. microspermum* (1,355,623, 3.3%) and *S. vermifera* (430,732, 1%). The respective alignments in lateral roots were considerably lower, *L. bicolor* with 1,001,193 aligned reads comprising 2.5%, *P. involutus* (424,155, 1%), *P. microcarpum* (213,166, 0.46%) and *S. vermifera* (318,539, 0.53%). Fungal gene expression analysis was thus possible only for the colonised root samples with *L. bicolor* and *P. involutus,* and for this purpose, we considered the control samples of free living mycelium grown on MMN agar medium by Shah et al. [79] and with following accession numbers: *L. bicolor* SRR1752511, SRR1752510 and SRR1752509, and *P. involutus* SRR1752470, SRR1752472 and SRR1752473. The RNA seq analysis pipeline was according to the oak RNA-seq analysis. Illumina reads from colonized roots and from vegetative mycelium were aligned against *L. bicolor* or *P. involutus* genome by TopHat2, gene expression levels were quantified by featureCounts, and the significance of differences in gene expression was assessed using edgeR. The thresholds for differential expression were fold change > 2 and Benjamini-Hochberg adjusted *P* < 0.01. Gene expression analysis was not performed for *P. microcarpus* and *S. vermifera* due to the low levels of fungal reads in colonized roots.

### Primer design and validation by qRT-PCR

To validate the differential expression of genes revealed by Illumina RNA sequencing, the expression of 12 of those genes was quantified by qRT-PCR in leaf samples from control and *L. bicolor*-inoculated plants. The same original RNA samples were used for cDNA synthesis for sequencing experiments as for qRT-PCR analyses. Among these 12 genes some were affected by all four fungi (*Qrob_P0407070.2* and *Qrob_P0502610.2*), others by the three EMF (*Qrob_P0403240.2),* or by three fungi (*L. bicolor*, *S. vermifera* and *P. involutus* or *P. microcarpus; Qrob_P0412360.2, Qrob_P0286850.2, Qrob_P0758050.2, Qrob_P0112730.2, Qrob_P0457340.2, Qrob_P0656320.2* and *Qrob_P0491740.2*), and two specifically by *L. bicolor* (*Qrob_P0135910.2* and *Qrob_P0273540.2*)*.* Primer pairs were constructed using the oak genome [12] as a reference and tested for functionality, amplicon size, specificity (single peak produced for each primer after melting curve analysis) and efficiency (using standard curve with dilution series of cDNA as template), and the qRT-PCR amplifications were performed according to Tarkka et al. [23]. Sequences of constructed primer pairs are listed in Additional file 14: Table S9. Briefly, using an iScript One-Step RT-PCR Kit with SYBR Green (Bio-Rad) and 18S rRNA as the reference gene, transcript abundances in the leaf samples were determined based on their Ct values using the Relative Expression Software Tool (REST, [80]). The coefficient of variation was used as a reproducibility indicator, with a maximal value of 6.0. Differential gene expression was determined by a randomization test implemented in REST.

## Supplementary information

**Additional file 1:Figure S1.** Influence of mycorrhizal fungi on oak growth.

**Additional file 2:Figure S2.** Numbers of reads obtained from samples under indicated treatments after sequence processing.

**Additional file 3:Table S1.** List of the differentially expressed genes in colonized roots for *L. bicolor* and *P. involutus*.

**Additional file 4:Figure S3.** Differential *L. bicolor* and *P. involutus* gene expression between colonized oak roots and free-living mycelium.

**Additional file 5:Table S2.** Plant genes differentially expressed after inoculation with the three EMF in colonized roots.

**Additional file 6:Table S3.** Differentially expressed hormone-related genes identified in colonized roots, non-colonized roots and leaves.

**Additional file 7:Table S4.** Most strongly differentially expressed plant genes after inoculation with all four fungi in non-colonized roots.

**Additional file 8:Table S5.** GO-terms identified among differentially expressed genes in non-colonized roots.

**Additional file 9:Figure S4.** Differential expression of disease-resistance gene families.

**Additional file 10:Table S6.** Differentially expressed defense-related genes.

**Additional file 11:Table S7.** Differentially expressed genes in leaves.

**Additional file 12:Figure S5.** Comparison of Illumina RNA-sequencing and qRT-PCR results for 12 selected genes differentially expressed in leaf samples of *L. bicolor*.

**Additional file 13:Table S8.** Putative mobile mRNAs corresponding to genes differentially expressed after inoculation with each of the fungi.

**Additional file 14:Table S9.** List of the primers used for real-time qRT-PCR analysis.

## Data Availability

Raw sequence data are available in the Sequence Read Archive at the National Center for Biotechnology Information (BioProject accession PRJNA516042).

## Abbreviations

DEG: Differentially expressed gene
ECM: Ectomycorrhiza
EMF: Ectomycorrhizal fungus
GO-term: Gene ontology term
MMN medium: Melin Norkrans modified medium
MMN1/10: Melin Norkrans modified medium with 1/10 P and N.
MMNC medium: Melin Norkrans modified medium supplemented with 0.1% (w/v) casein hydrolysate
NB-LRR protein: Nucleotide-binding site leucine-rich repeat protein
OMF: Orchid mycorrhizal fungus
RLK: Receptor-like kinase
