# Supplementary figures and images for: Oak displays common local but specific distant gene regulation responses to different mycorrhizal fungi
Source: BMC Genomics. 2020 Jun 12;21:399. doi: 10.1186/s12864-020-06806-5 (PMC7291512; doi:10.1186/s12864-020-06806-5)

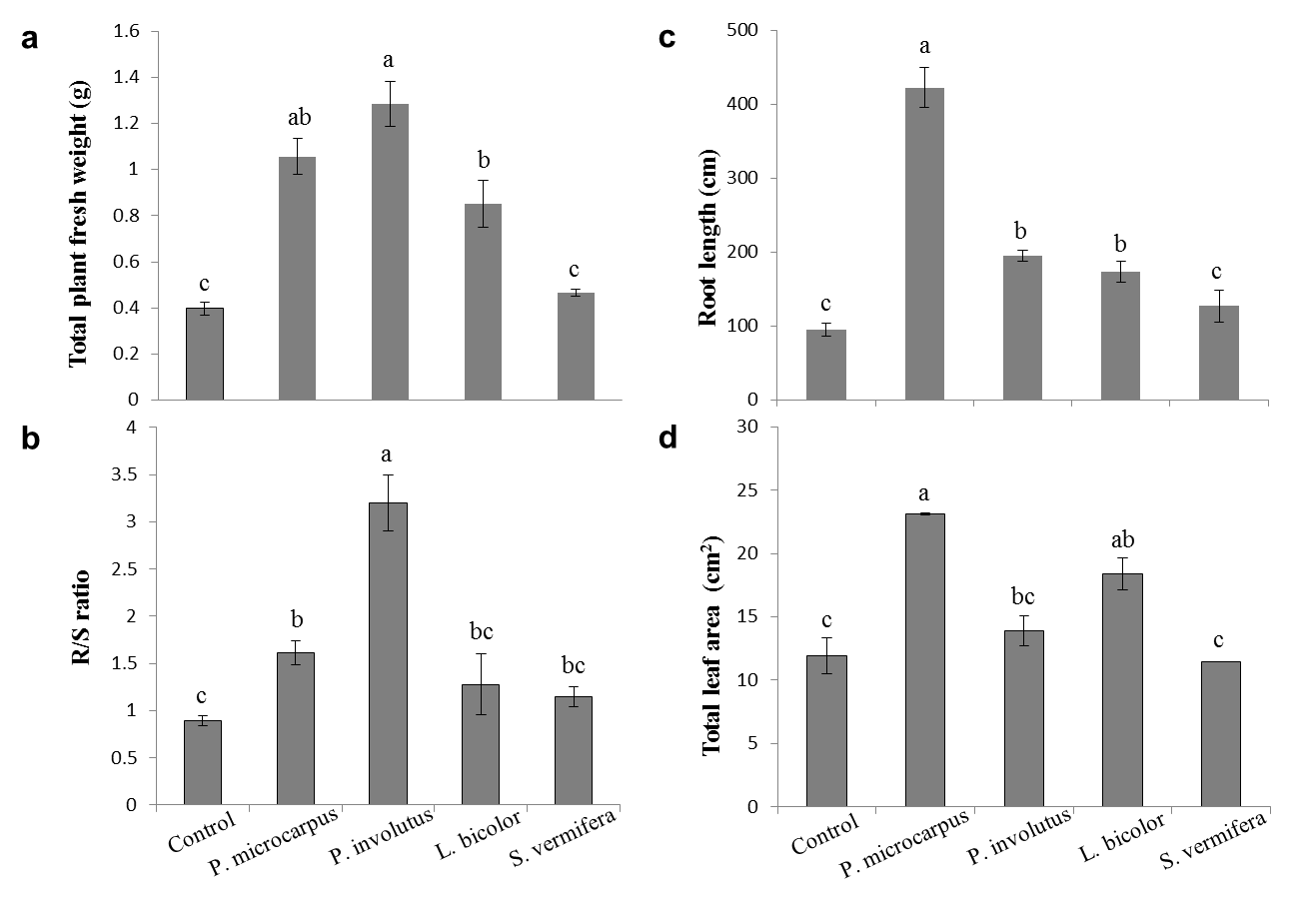

Supplement: Supplementary file 1 — Additional file 1:Figure S1. Influence of mycorrhizal fungi on oak growth. [file 12864_2020_6806_MOESM1_ESM.tif]

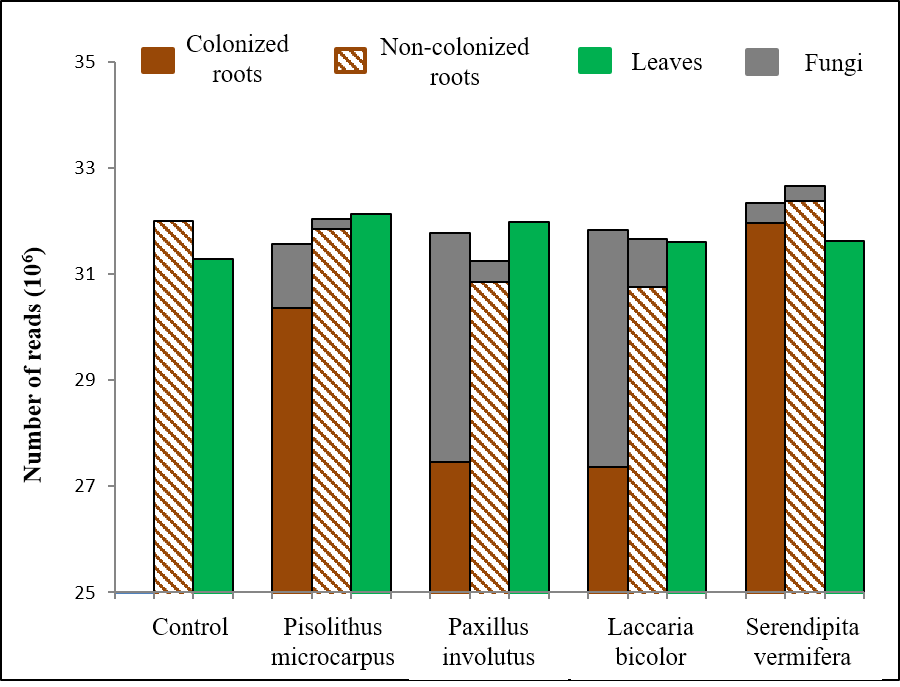

Supplement: Supplementary file 2 — Additional file 2:Figure S2. Numbers of reads obtained from samples under indicated treatments after sequence processing. [file 12864_2020_6806_MOESM2_ESM.tif]

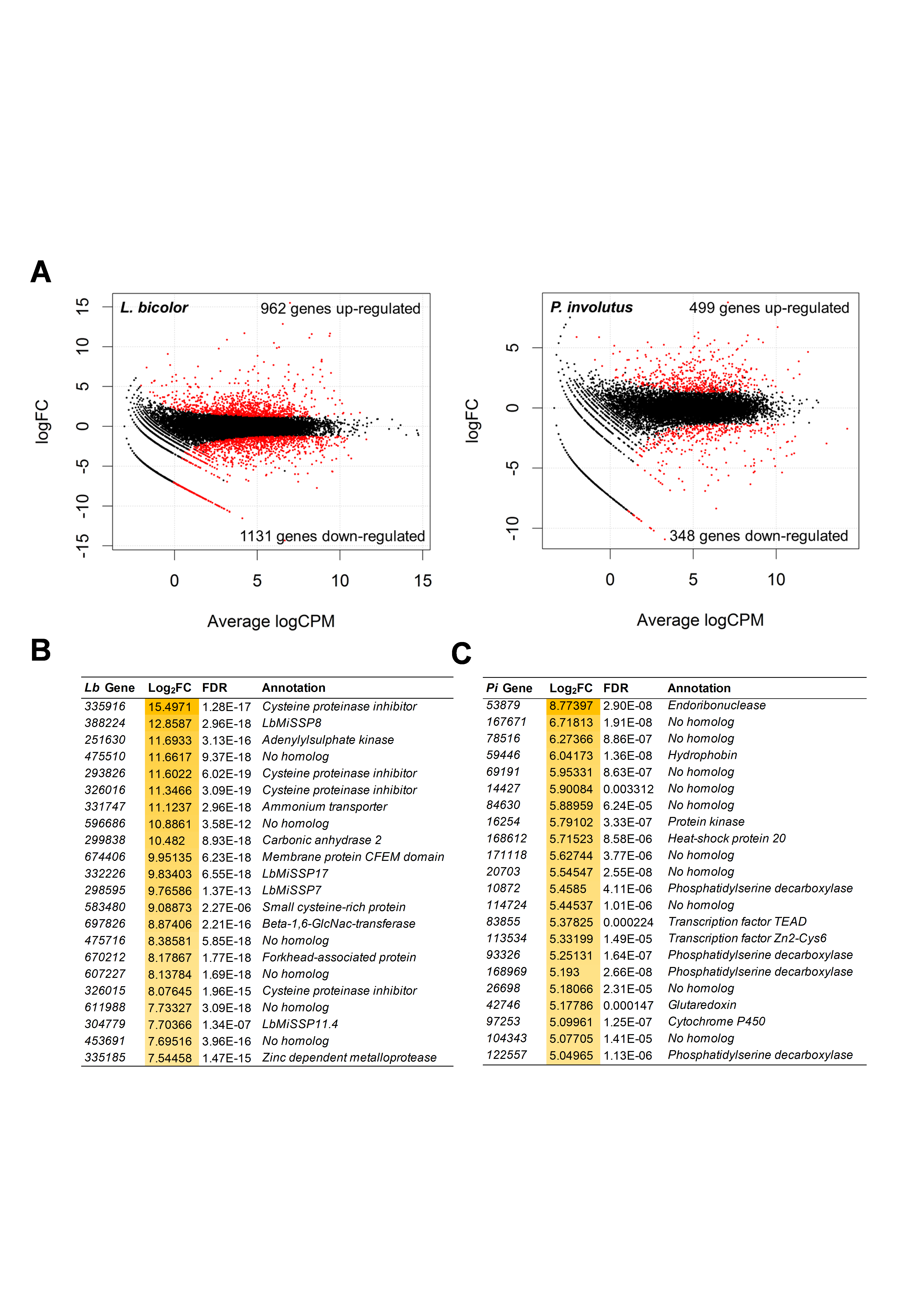

Supplement: Supplementary file 4 — Additional file 4:Figure S3. Differential L. bicolor and P. involutus gene expression between colonized oak roots and free-living mycelium. [file 12864_2020_6806_MOESM4_ESM.tif]

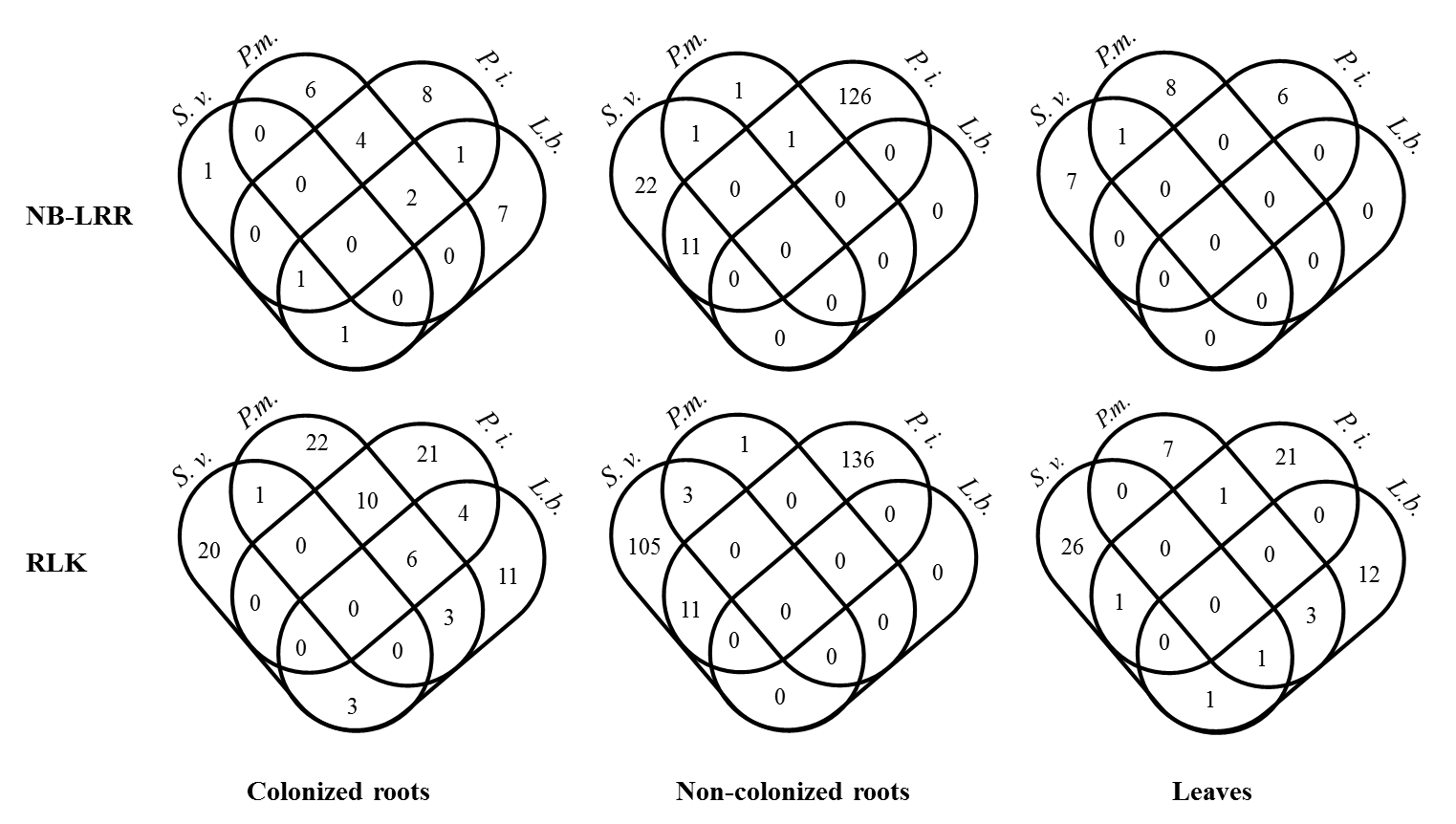

Supplement: Supplementary file 9 — Additional file 9:Figure S4. Differential expression of disease-resistance gene families. [file 12864_2020_6806_MOESM9_ESM.tif]

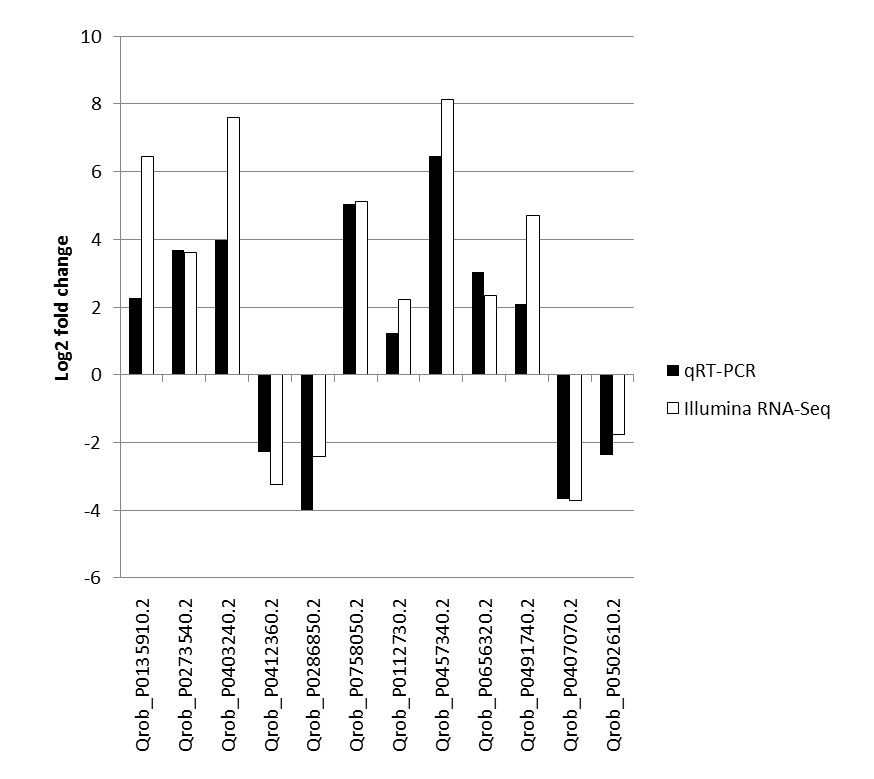

Supplement: Supplementary file 12 — Additional file 12:Figure S5. Comparison of Illumina RNA-sequencing and qRT-PCR results for 12 selected genes differentially expressed in leaf samples of L. bicolor. [file 12864_2020_6806_MOESM12_ESM.tif]
